# N-acetyl-L-cysteine functionalized nanostructured lipid carrier for improving oral bioavailability of curcumin: preparation, *in vitro* and *in vivo* evaluations

**DOI:** 10.1080/10717544.2017.1391890

**Published:** 2017-10-24

**Authors:** Cihui Tian, Sajid Asghar, Yifan Wu, Daddy Kambere Amerigos, Zhipeng Chen, Mei Zhang, Lining Yin, Lin Huang, Qineng Ping, Yanyu Xiao

**Affiliations:** a Department of Pharmaceutics, State Key Laboratory of Natural Medicines, China Pharmaceutical University, Nanjing, PR China;; b Faculty of Pharmaceutical Sciences, Government College University Faisalabad, Faisalabad, Pakistan;; c Faculty of Pharmaceutical Sciences, Department of Pharmaceutics and Drug analysis, University of Kinshasa, Kinshasa, Democratic Republic of the Congo;; d Department of Pharmacy, Nanjing University of Chinese Medicine, Nanjing, PR China

**Keywords:** N-acetyl-L-cysteine-polyethylene glycol (100)-monostearate, curcumin, nanostructure lipid carrier, oral delivery, intestinal mucus layer

## Abstract

The application of orally administered nanoparticles in the circulation system is limited by the secretion and shedding of intestinal tract mucous layer. In order to enhance mucoadhesion and mucus penetration of curcumin (Cur)-loaded nanostructured lipid carrier (NLC) after oral administration, a new multifunctional conjugate, N-acetyl-L-cysteine-polyethylene glycol (100)-monostearate (NAPG), was synthesized. Functionalized nanocarriers (Cur-NAPG-NLC) modified by different amounts of NAPG (the amounts of NAPG were 20, 50, and 100 mg) were prepared and investigated for *in vitro* and *in vivo* behavior. Mean particle sizes of 89–141 nm with negative zeta potential (−15 to −11 mV) and high encapsulation efficiency (EE, >90%) possessing spherical and stable nanocarriers were observed. Sustained drug release was also observed for the NAPG-NLC. *In situ* intestinal perfusion studies showed that with increasing the amount of NAPG increase absorption of Cur. *In vivo* oral pharmacokinetic evaluation suggested that the bioavailability of Cur in rats was proportional to the degree of functionalization of NLCs with NAPG. AUC_0–t_ of Cur-NAPG100-NLC was improved by 499.45 and 116.89 folds as compared to that of Cur solution and unmodified Cur-NLC, respectively. In conclusion, NAPG modified NLC could be a promising drug delivery system for improving oral performance of BCS class IV drugs.

## Introduction

Among various routes of drug delivery, oral administration is the preferred route owing to its ease of administration for patients (Balimane et al., 2000). Yet, many potential drug candidates are not suitable for oral route due to the efflux by P-glycoprotein, poor stability, low solubility, and permeability in GI tract (Ensign et al., 2012; Luo et al., 2014a). Nanoparticles have been a longstanding interest as an effective delivery system to enhance the oral bioavailability of these drugs. Among them, lipid based nanoparticles have been described as the most potential platform for lipophilic and poor water solubility drugs. Solid lipid nanoparticles (SLN), only containing solid lipid, have a series of prominent features, such as physical stability, controlled drug delivery, improved solubility, biocompatibility, and low cytotoxicity (Müller et al., 2002a; Müller et al., 2002b; Selvamuthukumar & Velmurugan, 2012). However, there is limited utilization of SLNs due to drug expulsion during storage and low drug loading capacity (Lancelot et al., 2014). Nanostructured lipid carriers (NLC) are developed because the liquid and solid lipid mixture forms the matrix of NLC, which prevents drug expulsion during storage and increases drug loading capacity (Hu et al., 2005).

The mucus layer on gastrointestinal (GI) tract which protects epithelial surfaces have been considered as the main challenge for oral nanoparticles, since its’ rapid secretion and clearance rate removes foreign particles (Lai et al., 2009; Ensign et al., 2012). Reports have shown that particles densely coated with highly hydrophilic surface can readily diffuse through mucus (mucus-penetrating particles) (Lai et al., 2009). Yang et al. (2014) discovered that polyethylene glycol (PEG) coating provided an uncharged and hydrophilic surface which could effectively minimize the adhesive interaction between mucin and nanoparticles, thus, the particles could rapidly diffuse through intestinal mucus. It was reported that the geometric mean effective diffusivity of PEG coated polymeric nanoparticles penetrating cystic fibrosis sputum was approximately 100-fold increase compared to the conventional polystyrene particles (Suk et al., 2009; Tang et al., 2009). Moreover, mucoadhesive nanoparticle systems which improve particles’ residence time in the GI tract and enhance drug absorption also attract the interest of researchers (Lai et al., 2009). Thiomers or so-called thiolated polymers can increase mucoadhesion by forming disulfide bonds with cysteine of mucus glycoproteins and also have a strong permeability to improve the absorption of drugs through the membrane (Bernkop-Schnürch et al., 2004b; Werle & Hoffer, 2006; Bravo-Osuna et al., 2007; Gradauer et al., 2012). The mucoadhesive properties of chitosan-4-thio-butylamidine conjugate showed almost 30 times improvement when compared to the unmodified chitosan (Bernkop-Schnürch et al., 2004a) and another research showed that the bioadhesion of chitosan-thioglycolic acid copolymer was increased by about 3–9 folds compared with the original chitosan (Kafedjiiski et al., 2005). Kast & Bernkop-Schnürch (2001) prepared chitosan thioglycolic acid conjugate (CS-TGA) which displayed thiol groups were about 6–39 μmol/g and the adhesion of these CS-TGA conjugates were approximately 6- to 10-fold greater compared to the unmodified polymer. N-acetyl-L-cysteine (NAC) is a small molecule with free thiol groups and has various pharmacological activities such as the treatment of congestive-obstructive pulmonary diseases and replenish cellular reduced glutathione (GSH) as precursor of amino acid cysteine (Moldéus & Cotgreave, 1994; Samuni et al., 2013). Furthermore, Khan et al. (1999) have found that NAC can disrupt the mucus barrier and cause a 6-fold increase of the absorption of polystyrene particles in rat intestine model. Lian et al. (2013) developed NAC functionalized chitosan-vitamin E succinate copolymer which enhanced the plasma concentration of paclitaxel 1.42-fold in comparison with that of paclitaxel solution after oral administration.

Curcumin (Cur) is a natural phenolic compound isolated from the rhizome of turmeric plant and possesses diverse biological activities, such as, antioxidant, anti-inflammatory, anticancer, antimalarial, and also has pharmacological activities against diabetes and Alzheimer’s disease (Nandakumar et al., 2006; Mishra et al., 2009; Nayak et al., 2010; Naksuriya et al., 2014). Unfortunately, the real therapeutic potential of Cur has not been fully utilized due to its low bioavailability which may result from its poor solubility (11 ng/mL in pH 5.0 buffer) (Tønnesen, 2002) and poor pharmacokinetic profile (the concentration of Cur in plasma was less than 5 ng/mL in rats after oral administration of Cur at a dose of 1 g/kg) (Wahlström & Blennow, 1978). Most of the studies focused on the nanoparticle delivery systems to improve the oral bioavailability of Cur. For example, the oral bioavailability of Cur in organogel-based emulsion was improved by 9 fold compared with unformulated Cur (Yu & Huang, 2012). Cur loaded PLGA nanoparticles displayed at least 9-fold high oral bioavailability compared to Cur Shaikh et al. (2009).

In this study, PEG and NAC co-modified NLC with mucus penetration and mucoadhesion mechanism was developed to promote oral absorption of Cur (Cur-NAPG-NLC). Since polyethylene glycol (100)-monostearate (S100) possessed the amphipathy, NAC was firstly conjugated to S100 to form N-acetyl-L-cysteine-polyethylene glycol (100)-monostearate (NAPG) and then three levels of NAPG modified Cur-loaded NLC (the amounts of NAPG was 20, 50, and 100 mg) were prepared by solvent evaporation method and characterized for particle size, zeta potential, encapsulation efficiency (EE), and surface PEG densities and distance. Appearance under transmission electron microscopy, X-ray diffraction, and *in vitro* release behavior were also investigated. The intestinal absorption of different Cur formulations were investigated by *in situ* single-pass perfusion and visualized by confocal laser scanning microscopy. In addition, their pharmacokinetics were also studied in rats after gavage administration.

## Materials and methods

### Materials

Curcumin (Cur, purity, 98.0%) was purchased from Aladdin Reagent Co., Ltd. (Shanghai, China); Polyethylene glycol (100)-monostearate (S100, the polymerization degree of ethylene glycol is 100) was purchased from Chenrun Chem Co., Ltd. (Nantong, China); N-acetyl-L-cysteine (NAC, purity, 98.0%) was purchased from Yuanye Bio-Technology Co., Ltd. (Shanghai, China); Cholesterol oleate (purity, >85%) was purchased from Tokyo Chemical Industry (Tokyo, Japan). Glycerol trioleate (purity, >98%) and phosphatidylcholine (PC, purity >90%) were kindly donated by Evonik Degussa China Co., Ltd. (Shanghai, China). Coumarin-6 (C6) was purchased from Tokyo Chemical Industry (Tokyo, Japan); 1-3-dimethylaminopropyl-3-ethylcarbodiimide hydrochloride (EDCI) was purchased from the Energy Chemical (Shanghai, China); 3-4,5-dimethylthiazol-2-yl -2,5-diphenyltetrazolium bromide (MTT) was purchased from Sigma-Aldrich (Milwaukee, WI). All other chemicals and reagents were of analytical grade and used without further purification.

### Animals

Male Sprague–Dawley (SD) rats (180–220 g) and ICR mice (18–22 g) were obtained from Experiment Animal Center of Nantong University (Nantong, China). All animal experiments were approved by Institutional Animal Care and Use Committee of China Pharmaceutical University.

### Synthesis of N-acetyl-L-cysteine-polyethylene glycol (100)-monostearate (NAPG)

NAPG was synthesized according to a previous research with some modifications (Meng et al., 2015). Briefly, NAC (1 mmol) was dissolved in anhydrous N,N-dimethylformamide (5 mL) and then 0.63 mmol EDCI was added. This reaction was continued for 1 h in an ice bath. Afterwards, S100 (0.43 mmol) was dissolved in CH_2_Cl_2_ (25 mL) and slowly added to the above solution, which was first stirred for 3 h in an ice bath and then stirring was continued for 72 h at room temperature. Subsequently, the organic solvent was removed using a rotary evaporator. The resultant solution was dialyzed with a dialysis bag (3.5 kDa MW cutoff, Sigma-Aldrich, Milwaukee, WI) at room temperature for 48 h to remove the unreacted NAC and lyophilized to obtain NAPG. The chemical structure of NAPG was analyzed by ^1^H-NMR spectra (AVANCE AV-300, Bruker Instrument Inc., Switzerland) with D_2_O as the solvent at 25 °C. Furthermore, surface tension measurements (Shanghai Shibo. Instrument Co. Ltd., Shanghai, China) were also done to measure the critical micelle concentration (CMC). Briefly, S100-NAPG and S100 solutions (0.001–1.5 mg/mL) were prepared. The surface tension of solutions was measured and plotted against the logarithm of sample concentration; the CMC was determined by taking the cross-point when extrapolating the surface tension.

### Determination of coupling ratio of NAC on NAPG

The amount of NAC in NAPG conjugate was determined according to Pharmacopoeia of People’s Republic of China with little modification (Pharmacopoeia Committee of P. R. China, 2015). This method is based on oxidation reduction titration. Iodine, as an oxidizing agent, reacts with the thiols present in NAC. In brief, about 0.1 g NAC was dissolved in 10 mL water and immediately titrated with 0.05 mol/L iodine solution until a pale yellow color was observed and persisted for 30 s (the volume of the iodine solution was defined as V_0_). Accurately weighed 4.8 g of NAPG was dissolved in 10 mL water. The above mentioned method was repeated and the volume of the iodine solution was defined as V_1_. The coupling ratio (CR) was calculated with the following equation:

$$\text{CR }\left(\%\right)=\;\left(\frac{M_{\text{NAPG}}\times V_{1}\times W_{\text{NAC}}}{M_{\text{NAC}}\times V_{0}\times W_{\text{NAPG}}}\right)\times \text{1}00$$

where *M*_NAPG_ is the molecular weight of NAPG, *W*_NAC_ is the weight of NAC added, *M*_NAC_ is the molecular weight of NAC, and *W*_NAPG_ is the weight of NAPG added.

### Preparation of Cur-NAPG-NLC

Cur-loaded NLC (Cur-NLC) was prepared by a solvent evaporation method as described by Meng et al. (2015). Briefly, lipid matrix materials (330 mg of PC, 20 mg of cholesterol oleate, 70 mg of glycerol trioleate) and Cur (16 mg) were dissolved in 10 mL of ethanol-chloroform mixture (1:1, v/v) and dried in a rotary evaporator under vacuum at 40 °C for 4 h. The dried lipid film was hydrated with 10 mL 10% (v/v) glycerol solution at 37 °C for 30 min, then ultrasonicated at 300 W for 5 min, followed by extrusion through 0.45 µm cellulose nitrate membrane to remove the unentrapped Cur and obtain Cur-NLC. For the preparation of NAPG modified NLC, different amounts of NAPG (20, 50, and 100 mg) were added into the lipid matrix, and then the same procedures was adopted as described above. Correspondingly, based on the amounts of NAPG, the NAPG modified NLCs were named as Cur-NAPG20-NLC, Cur-NAPG50-NLC, and Cur-NAPG100-NLC. Blank NAPG-NLC was prepared as mentioned above only without Cur.

### Characterization of cur-NAPG-NLC

#### Particle size, zeta potential, morphology, encapsulation efficiency (EE), and drug loading content (DL)

The particle size, polydispersity index (PDI), and zeta potential of Cur-NLC and different Cur-NAPG-NLC (dispersed in deionized water) were detected by Zeta Plus (Brookehaven, New York, NY). All measurements were taken in triplicate. The morphologies of Cur-NLC and Cur-NAPG50-NLC (as a representative of Cur-loaded NAPG-NLC) were observed under transmission electron microscopy (TEM, H-7000, Hitachi, Japan). The Cur content in the drug-loaded NLC was measured via HPLC system (Shimadzu LC-10AT HPLC system, Tokyo, Japan). C_18_ column (5 μm, 150 mm × 4.6 mm) was kept at 40 °C with the mobile phase of 5% (v/v) acetic acid in purified water: acetonitrile =50:50 (v/v). The injection volume was set at 20 μL. The flow rate was 1.0 mL/min and the effluent was monitored at 420 nm. Cur-NLC and different Cur-NAPG-NLC were filtered through the 0.45 μm cellulose nitrate membrane. The 100 μL filtrate was mixed with 900 μL methanol and vortexed for 30 s, and then centrifuged at 12,000 rpm for 10 min. The amount of drug before and after filtration was determined. The EE and DL were calculated from following equations (Yang et al., 2015):

$$\text{EE}\left(\%\right)=\left(\frac{\text{Weight of Cur in NLC}}{\text{Weight of the initial Cur}}\right)\times \text{1}00\%$$

$$\text{DL}\left(\text{\%}\right)\text{=}\left(\frac{\text{Weight of Cur in NLC}}{\text{Weight of NLC}}\right)\times \text{1}00\%$$

#### X- ray diffraction (XRD) study

The crystalline properties of the samples were analyzed using D8 Advance X-ray Diffractometer (Bruker, Germany). XRD patterns were determined for cholesterol oleate, Cur, physical mixture of Cur and NAPG50-NLC, blank NAPG50-NLC and Cur-NAPG50-NLC (as the representative of blank NAPG-NLC and Cur-loaded NAPG-NLC, respectively) with a Cu Ka radiation (*k* = 1.5406 Å) under 40 kV voltage and 40 mA current. The scans were carried out at 3–40° over 2θ with a count time and a step angle of 0.3 s and 0.02°, respectively.

#### Differential scanning calorimetry analysis (DSC)

DSC was used to analyze thermal properties of NAPG, Cur, blank NAPG50-NLC, and Cur-NAPG50-NLC. They were sealed in 40 µL aluminum pans and curves were recorded with a scan rate of 10 k/min in the 50–300 °C temperature range using DCS-60 (Shimadzu, Kyoto, Japan).

#### Surface densities and distance of PEG in the NAPG-NLC

Taking advantage of the formation of a complex between PEG and iodine, which has a specific color reaction, and then there is a specific absorption at 500 nm and a linear relationship with the content of PEG, a colorimetric method with slight modifications was used for the quantification of the NAPG. The amount of non-adhered NAPG (W_1_) was obtained by ultrafiltration with molecular weight cutoff 100 kDa and determined by colorimetric method described by Su et al. (2011) with a slight modification. Then, the concentration of the non-adhered NAPG was calculated from a standard curve in a range from 2.5 to 100 μg NAPG/mL. The amount of total NAPG in NAPG-NLC (W_2_, including the adhered and non-adhered NAPG on the surface of modified NLC) was directly measured without ultrafiltration. The amount of adhered NAPG on the surface of NAPG-NLC (W_PEG_) was equal to (W_2_−W_1_). The surface density of PEG chains (SD_PEG_) and distance between PEG chains on the surface of Cur-NAPG-NLC (D_PEG_)were calculated by the following equation (Su et al., 2011).

$${\text{SD}}_{\text{PEG}}=\frac{N_{\text{PEG}}}{S_{\text{NP}}}$$

$$D_{\text{PEG}}={\left(\frac{\text{1}}{{\text{SD}}_{\text{PEG}}}\right)}^{\text{1}/\text{2}}$$

where *N*_PEG_ is the total number of PEG chains, and *S*_NP_ is the surface area of the NLC. Resolving, *N*_PEG_ = (*W*_PEG_ / Molecular weight of NAPG) ×Avogadro’s number. *S*_NP_ = 4 *πr*^2 ^×*N*_NP_, where *r* is the particle radius and *N*_NP_ is the total number of nanoparticles was calculated as:

$$N_{\text{NP}}=\frac{M_{t}}{M_{\text{s}}}$$

where *M_t_* is the total weight of NLC, which can be obtained by lyophilizing the NLC suspension (NLC suspension was frozen at −20 °C for 4 h followed by −80 °C for 12 h and then dried under vacuum for 48 h) and weighing, *M*_s_ is the mass of one particle. Resolving, *M*_s_ = *ρ*×*V* = *ρ* × 4 *πr*^3^/3, where *ρ* is the density of NLC suspension, and the value is approximately equal to water’s density, *V* is the volume of one particle, and *r* is the radius of NLC.

### *In vitro* release

*In vitro* release of Cur from NLC and different NAPG-NLC were studied using dialysis bag diffusion technique. pH 1.2 hydrochloric acid solution, pH 6.8 PBS, and physiological saline were used as release media. For meeting sink conditions, 1% (w/v) tween 80 was added to the above media. Typically, 1 mL of the sample (containing 400 µg of Cur) was poured into dialysis bags (8–10 kDa MW cutoff, Sigma-Aldrich, Milwaukee, WI). Cur solution (1.5 mg of Cur dissolved in 10 mL of polyethylene glycol 400, and then diluted to 50 μg/mL with deionized water) was taken as a control. The bags were suspended in 50 mL of release medium with shaking (100 rpm) at 37 ± 0.5 °C. Then, 1 mL of medium was withdrawn at desired time intervals and 1 mL of pre warmed fresh medium was added to maintain the volume. The concentration of Cur was analyzed by HPLC as described above. The time and medium used were the following: (1) 0–2 h in the pH 1.2 hydrochloric acid solution and 2–24 h in the pH 6.8 PBS; (2) 0–24 h in the physiological saline.

### *In situ* single-pass intestinal perfusion study

The Cur intestinal circulating perfusion solution (equivalent to 40 µg/mL of Cur) was prepared by diluting Cur solution, Cur-loaded NLC and NAPG-NLC with Krebs-Rings (KR) buffer solution.

The absorption of Cur solution, Cur-loaded NLC and NAPG-NLC in rats intestinal tract were investigated based on the established method with slight modifications (Dezani et al., 2016). Briefly, male SD rats were fasted 12 h and allowed to access water freely before experiment. Rats were anesthetized with intraperitonial injection of 3% chloral hydrate (480 mg/kg) and placed under an infrared light to maintain a normal body temperature. Intestinal segments (10 cm long of duodenum, jejunum, and ileum) were carefully cannulated at both ends. Then, they were rinsed with physiological saline (37 °C) which was pumped by peristaltic pump (HL-2, HUXI, China) through the intestine at a flow rate of 0.5 mL/min until the residual debris were cleaned out, and intestinal segments were equilibrated with KR buffer solution at the same flow rate for 30 min. After that, the intestine was perfused by Cur perfusion solution at a flow rate of 0.2 mL/min. Each perfusion experiment lasted for 120 min and samples were collected at an interval of every 15 min in a pre-weighed glass tube. Then, 900 μL of methanol was added into 100 µL of perfusion solution sample, vortexed for 30 s and centrifuged at 12,000 rpm for 10 min. Twenty µicroliter of supernatant was injected into the HPLC for Cur determination. Finally, the rats were sacrificed, and the radius (*r*), and length (*l*) of each intestinal segment were measured.

The intestinal absorption rate (ka) and the effective permeability coefficient (*P*_eff_) were calculated as indicated below:

$$\text{Ka}=\;\left(\frac{\text{1}-C_{\text{out}}\times Q_{\text{out}}}{C_{\text{in}}\times Q_{\text{in}}}\right)\times \frac{Q}{\pi r^{\text{2}}l}$$

$$P_{\text{eff}}=\;\frac{\left[-Q\times \text{ln}\left(\frac{C_{\text{out}}\times Q_{\text{out}}}{C_{\text{out}}\times Q_{\text{out}}}\right)\right]}{\text{2}\pi rl}$$

Where *C*_out_ and *C*_in_ are outlet and inlet Cur concentration, respectively; *Q*_out_ and *Q*_in_ are outlet and inlet perfusate flux (mL/min), respectively; *r* is the intestinal radius (cm) and *l* is the intestinal length (cm).

### Distribution in the intestinal tract after oral administration of NLC and NAPG-NLC to ICR mice

To observe the distribution of different NLC in the intestinal tract, coumarin-6 (C6) -loaded NLC and NAPG-NLC were prepared as mentioned previously, but Cur was replaced with C6. ICR mice were intragastrically administered different C6-loaded NLC at a dose of 10 mg/kg of C6. After 120 min, the mice were sacrificed, and the duodenum, jejunum, and ileum sectioned at 20 µm and frozen in cryoembedding media (OTC) at −80 °C. After that, the frozen intestines were placed on glass slides and fixed with 4% formalin at 25 °C for 10 min. Finally, the coverslips were observed by CLSM (Leica TCS SP5).

### Pharmacokinetic profiles

SD rats were randomly divided into six groups (*n* = 6). Before the experiment, they were fasted for 12 h but allowed to take water freely. Cur solution, Cur-NLC, Cur-NAPG20-NLC, Cur-NAPG50-NLC, and Cur-NAPG100-NLC equivalent to 50 mg/kg of Cur were given to rats by intragastric administration, respectively. In order to assess the absolute bioavailability of formulations, Cur solution was given to rats by intravenous (i.v.) injection at dose of 2 mg/kg of Cur. Then, 0.5 mL of blood samples were collected from the eyeground veins at certain time points. The plasma samples obtained after centrifugation at 4000 rpm for 10 min were stored at −20 °C until analyzed. Two hundred microliter of acetonitrile was added into 100 µL of plasma, vortexed for 2 min and centrifuged at 12,000 rpm for 10 min. Twenty microliter of supernatant was injected into the HPLC with a fluorescence detector to determine the concentration of Cur. The excitation and emission wavelengths were 436 and 518 nm, respectively.

Peak concentration (*C*_max_) and peak time (*T*_max_) were directly obtained from the experimental points. The other pharmacokinetic parameters were computed by software program DAS version 2.0 (Medical College of Wannan, Anhui Sheng, China). The absolute bioavailability (F_Abs_) of oral formulations was determined by using the equation:

$$F_{\text{Abs}}=\;\left(\frac{{\text{AUC}}_{0-t\;(p.o.)}\times {\text{Dose}}_{i.v.}}{{\text{AUC}}_{0-t\;(i.v.)}\times {\text{Dose}}_{p.o.}}\right)$$

where AUC_0_*_–t (p.o.)_* and AUC_0_*_–t (i.v.)_* are the individual total area of CUR plasma concentration time profile after oral and i.v. administration of formulations and Cur solution (i.v.) at Dose*_p.o._* and Dose*_i.v._* equivalent to Cur, respectively.

### Statistical analysis

Results were given as mean ± SD. Statistical significance was tested by variance (ANOVA) or two-tailed Student’s t-test. Statistical significance was set at *p* < .05, and extreme significance was set at *p* < .01.

## Results and discussion

### Characterization of NAPG

**Figure 1.**
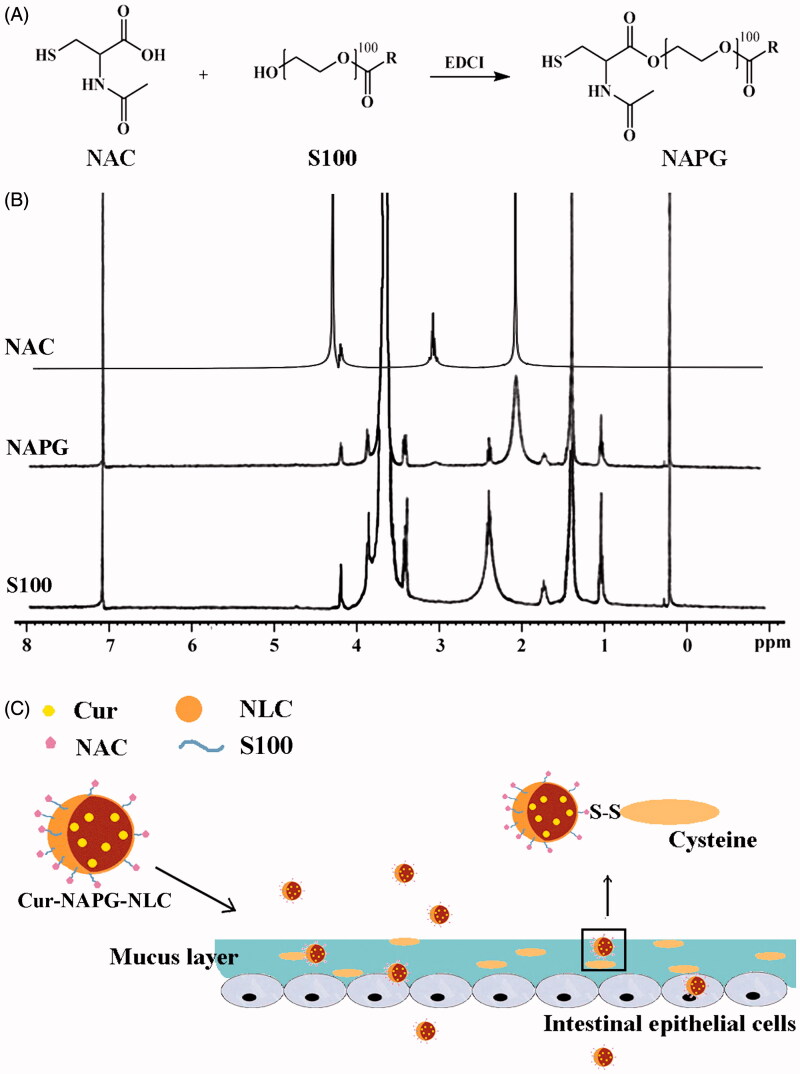
(A) Synthesis of NAPG; (B) ^1^H-NMR spectrum of NAC, S100, and NAPG in D_2_O at 25 °C; (C) schematic illustration of Cur-NAPG-NLC for oral delivery of Cur by the formation of disulfide bond between NAPG with cysteine in mucus layer and PEG coating.

NAC has free thiol groups that can form covalent S─S bonds with the mucus cysteine to obtain the steady adhesion of the nanomaterial to the mucus layer (Roldo et al., 2004; Makhlof et al., 2010). Therefore, in this study, we conjugated the carboxyl group of NAC with the hydroxyl groups of S100 in the presence of EDCI to obtain the new amphiphilic material (NAPG). The synthesis process of NAPG is shown in detail in Figure 1(A). Lyophilized NAPG was white, odorless, and fibrous. The structure of NAPG was confirmed by ^1^H-NMR (Figure 1(B)). Acetyl group of NAC at about 2.0 ppm was observed in NAPG, which indicates that NAC was successfully conjugated to the backbone of S100 to form NAPG. The CMC value of S100 and NAPG were 62.85 ± 0.11 and 25.82 ± 0.07 µg/mL, respectively, which revealed that the conjugation of NAC increased the amphiphilicity of S100. The mean CR of NAC on three batches of NAPG was 33.66 ± 0.95%.

### Preparation and characterization of Cur-NAPG-NLC

**Figure 2.**
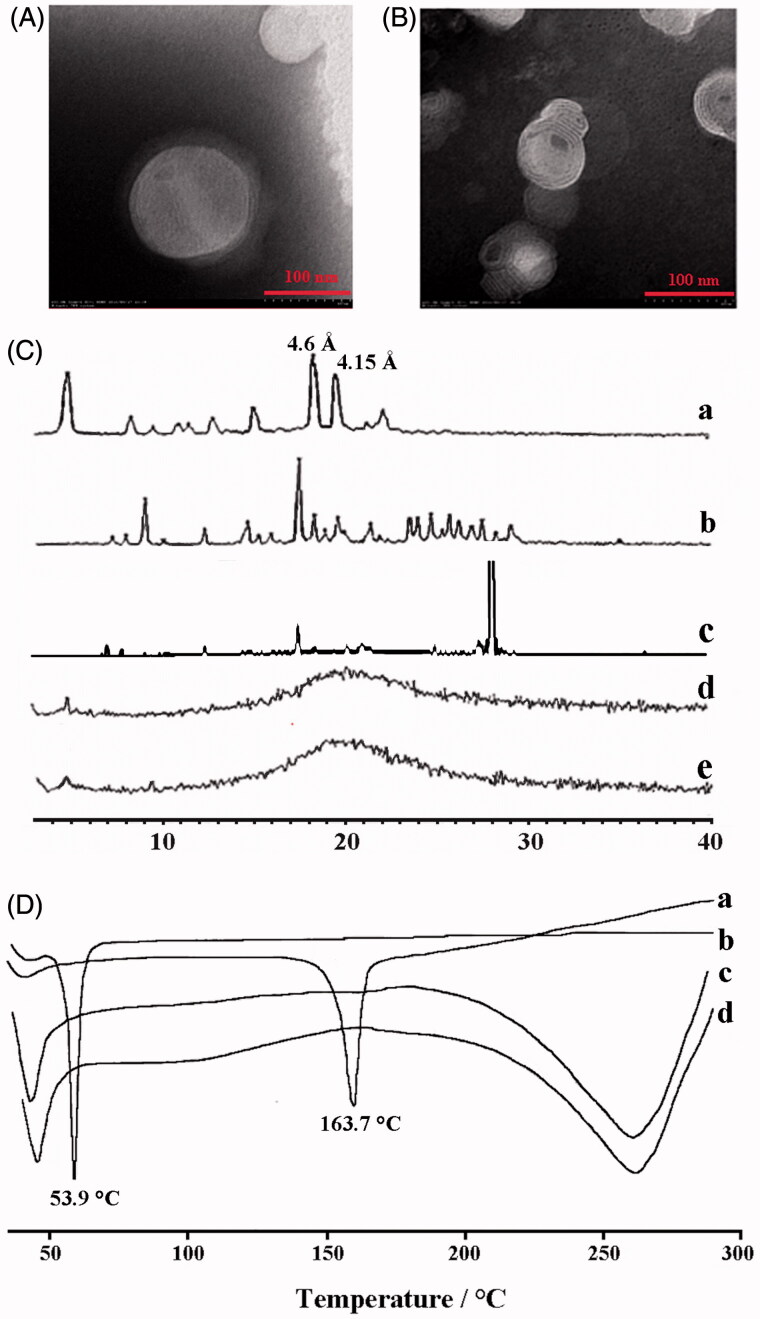
The TEM images of (A ) Cur-NLC and (B) Cur-NAPG50-NLC; (C) Power X-ray diffraction patterns of: Cholesterol oleate (a), Cur (b), physical mixture of Cur and NAPG50-NLC (c), blank NAPG50-NLC (d) and Cur-NAPG50-NLC (e); (D) DSC curves of: Cur(a), NAPG (b), blank-NAPG50-NLC (c) and Cur-NAPG50-NLC (d).

The schematic presentation of Cur-NAPG-NLC (Figure 1(C)) shows hydrophobic monostearate of NAPG was located inside the core of NLC, NAC, and PEG were located on the surface of NLC, and Cur was effectively encapsulated in the core of NLC. Cur-NAPG-NLC rapidly penetrated into the mucus layer and adhered to the mucus by the formation of the disulfide bond between free thiol groups of NAC and the mucus cysteine domain, and then was endocytosed by epithelial cells into the systemic circulation. The particle size, PDI, zeta potential, EE, and DL of NLC are listed in Table 1. Mean size of Cur-NLC was 141.2 nm, after modification with NAPG the particle size of NLC decreased to 121.1, 100.9, and 89.2 nm for Cur-NAPG20-NLC, Cur- NAPG50-NLC, and Cur- NAPG100-NLC, respectively. The modification of NAPG on the surface of NLC led to a decrease in particle size of NLC caused by the surface-active effect of NAPG (Lim & Kim, 2002). Furthermore, the surface PEG chains would have restricted the growth in the particle size (Liang et al., 2005). All formulations had a narrow size distribution as evident by lower PDI of below 0.3 (Luo et al., 2014b). The zeta potential of Cur-NAPG20-NLC was −13.52 mV, while it was observed that increase of NAPG decreased the absolute value of the zeta potential of NLC (the zeta potential of Cur-NAPG100-NLC was −9.55 mv), PEG on the surface of NLC might have partially masked the exposed negative moieties (Luo et al., 2015). It has been reported that more than −30 mV surface charge provides sufficient stability to the NLC (Lim & Kim, 2002). However, NAPG contains hydrophilic PEG and might impart an additional steric shield to the nanoparticles (Su et al., 2011). Consequently, electrostatic repulsion in tandem with the steric hindrance would ensure a stable dispersion. High EE (>90%) and about 3% of DL were observed in all the preparations. The quasi-spherical shapes of Cur-NLC and Cur-NAPG50-NLC were demonstrated by TEM images (Figure 2(A,B)). The results show that they nanoparticles were uniformly dispersed without any aggregation.

**Table 1.** Physicochemical properties of different NLC (mean ± SD, *n* = 3).

	Cur-NLC	Cur- NAPG20-NLC	Cur-NAPG50-NLC	Cur-NAPG100-NLC
Size (nm)	141.2 ± 3 .9	121.1 ± 3.1	100.9 ± 4.3	89.2 ± 1.5
PDI	0.17 ± 0.017	0.18 ± 0.04	0.20 ± 0.03	0.24 ± 0.098
Zeta potential (mV)	−13.96 ± 1.43	−13.52 ± 0.51	−11.95 ± 0.43	−9.55 ± 0.39
EE (%)	91.57 ± 0.87	93.31 ± 0.05	97.13 ± 0.02	95.68 ± 0.04
DL (%)	3.11 ± 0.54	3.07 ± 0.001	3.04 ± 0.003	2.98 ± 0.004
SD_PEG_	–	0.58 ± 0.049	1.61 ± 0.071	2.32 ± 0.019
D_PEG_ (nm)	–	1.32 ± 0.056	0.79 ± 0.006	0.66 ± 0.095

–no data.

The XRD analysis was employed to find out the amorphous or crystalline state of Cur in NAPG50-NLC and reveal the crystalline form of the lipid, which is particularly important for lipid based formulations as lipids usually demonstrate polymorphic transitions leading to drug expulsion and recrystallization. The XRD patterns of cholesterol oleate, Cur, blank NAPG50-NLC and Cur-NAPG50-NLC are shown in Figure 2(C). Figure 2(C) indicates that cholesterol oleate and Cur existed in a crystalline state before being processed to produce NAPG-NLC formulation and these were also observed in the patterns obtained for the physical mixture of Cur and NAPG50-NLC. Bragg-spacing values calculated were for the reflections of the crystalline state of the lipid. As explained by Melissa et al. (2015), the characteristic spacings of β form, β' form, and α form were at 4.6 , 3.8, and 4.15 Å, respectively. Figure 2(C) (a) shows existence of 4.6 and 4.15 Å Bragg-spacing values, which indicates that the cholesterol oleate used in our study was a mixture of β form and α form. The α form provides a homogeneous texture resulting in the formation of quite tiny crystal particles. The β form has the highest melting point and is the most stable (Marangoni & Narine, 2002). NAPG-NLC prepared with and without Cur exhibited a completely different peak with the starting lipid material and the intensity peaks that belong to Cur disappeared in the diffractograms of Cur-NAPG50-NLC, suggesting the amorphous form of the lipid formulations, as well as revealing crystalline drug was loaded into the lipid core in a solid solubilized state (Mohanty & Sahoo, 2010).

The DSC thermogram of pure Cur peaked at 163.7 °C (Figure 2(D)). However, the endothermic peak of Cur disappeared in Cur-loaded NAPG50-NLC, indicating the existence of amorphous Cur or that it has been molecularly dispersed within the lipid matrix (Yang et al., 2015). These results support the findings observed with XRD. In addition, the DSC thermograms shows that NAPG has a melting peak at 53.9 °C while the melting peak of NAPG50-NLC with or without Cur payload is below 53.9 °C. The amphiphilic molecule (NAPG) in the molten lipid may interact with the lipid core resulting in reduced energy required for melting (Fang et al., 2008).

#### *SD_PEG_ and D_PEG_ of PEG in the surface of Cur*-*NAPG-NLC*

PEG coatings not only provide dispersion stability for nanoparticles, but also shield their core from adhesive interactions with mucus (Walkey et al., 2012; Yang et al., 2014). SD_PEG_ of Cur-NAPG100-NLC (Table 1) was 4.00-fold and 1.44-fold higher than that of Cur-NAPG20-NLC and Cur-NAPG50-NLC, respectively. It is reported that molecular weight of PEG, degree of modification, and the size of the nanoparticles affect SD_PEG_ (Soong & Macdonald, 2007). As the amount of NAPG increased, higher was the resultant SD_PEG_. Increased SD_PEG_ hinders the binding of mucin to NLC surfaces and thus the transport of NLC in mucus could be much faster (Xu et al., 2015). Based on this conclusion, we speculate that NAPG100-NLC will have better *in vivo* performance than other formulations. D_PEG_ of Cur-NAPG20-NLC and Cur-NAPG50-NLC was 2.00-fold and 1.20-fold wider than that of Cur-NAPG100-NLC, respectively, indicating that two adjacent PEG grafting sites got narrower. Gref et al. had reported a radius of 2–5 nm for majority of the plasma proteins (Gref et al., 1995). Nanoparticles could resist the adsorption of the plasma proteins and escape the recognition by Mononuclear Phagocyte system (MPS) for the D_PEG_ below 2 nm (Su et al., 2013). Here, the D_PEG_ for all modifications was less than 2 nm which suggests that longer circulation time might occur for all the NAPG modified NLC.

### *In vitro* release

**Figure 3.**
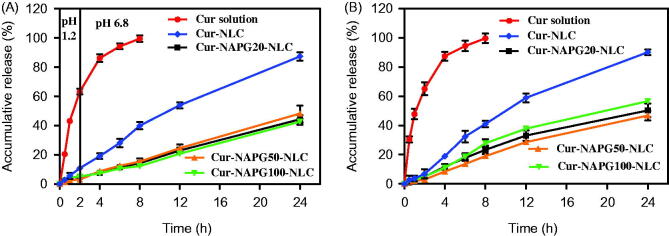
*In vitro* cumulative drug release profiles from Cur solution, Cur-loaded NLC and NAPG-NLC in (A) pH 1.2 hydrochloric acid solution for 2 h and in pH 6.8 PBS for next 22 h and (B) physiological saline containing 1% tween 80 at 37 °C (mean ± SD; *n* = 3).

The *in vitro* drug release characteristics of the Cur solution, Cur-loaded NLC and NAPG-NLC were determined in three-different pH release media (pH 1.2 hydrochloric acid solution, pH 6.8 PBS solution, and physiological saline). The *in vitro* release curves were obtained by plotting the cumulative percentage of Cur released with respect to the total amount of Cur encapsulated in the NLC or NAPG-NLC as a function of the time (Figure 3). In case of Cur solution, almost 80% of the total drug released within 4 h, while sustained release of Cur was observed for all NLC formulations. In the first 2 h in acidic medium, only 10 and 5% of the loaded Cur was released from the NLC and NAPG-NLC, respectively. The sustained release effect of NAPG modified NLC is better than that of NLC as PEG chains of NAPG might have limited the diffusion of the Cur from NLC (Su et al., 2014). Release of Cur from different NAPG-NLC yielded similar profiles. In pH 6.8 PBS solution and physiological saline, NAPG-NLC showed about 45–50% Cur release up to 24 h. The better control over the drug release in intestinal environment would allow to maintain good level of Cur in the blood for a prolonged time.

Various models were utilized to simulate the release profiles and the release kinetic parameters as shown in Table 1 of Supplementary material. According to the values of *R*^2^ and AIC, Weibull model was best fitted to explain the release mechanisms of Cur from NLC and NAPG-NLC. Papadopoulou et al. (2006) explained the drug release mechanism by interpretation of the exponent parameter (β) of Weibull model. Here, the β value of Cur-NLC and Cur-NAPG-NLC (except Cur-NAPG-NLC in physiological saline which followed the Fickian diffusion and Case II transport model) were above 1 which iterates that the Cur release from NLC or NAPG-NLC was a complex mechanism (a nonlinear increase in the release rate which decreases afterwards).

### *In situ* single-pass intestinal perfusion study

A comparison between the drug concentration before and after pumping system was used to verify the adsorption of Cur in components of pumping system. In addition, the physical absorption of the Cur was assessed by incubation at 37 °C for 2 h with intestinal segments. Samples were analyzed by HPLC system. In both cases, the change in drug concentration was less than 5%, which suggested that there was no loss of drug due to the adsorption of Cur in pump’s components and physical absorption by intestinal segments. Furthermore, histological evaluations of lumens were carried out at the end of perfusion. A normal intestinal epithelial tissue was observed which suggests that the formulations had good biocompatibility (data not shown).

**Figure 4.**
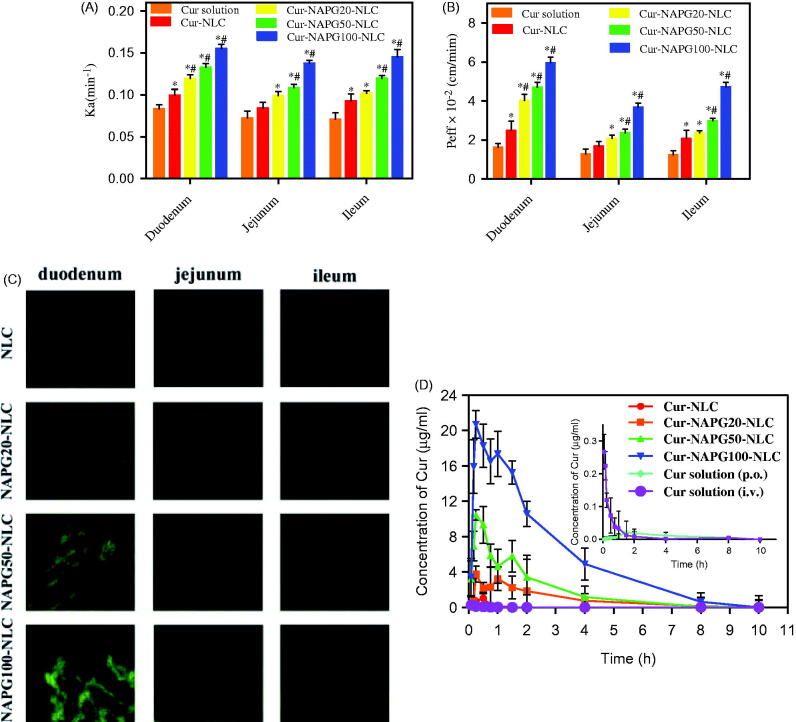
*In situ* single-pass perfusion, (A) Ka and (B) P_eff_ of duodenum, jejunum and ileum. *p < .05 versus Cur solution, #*p* < .05 versus Cur-NLC (mean ± SD; *n* = 3). (C) Distribution of C6 labeled different NLC in duodenum, jejunum, and ileum after oral gavage to mice by CLSM. (D) Plasma concentration–time profiles of Cur in rats after gavage administration of Cur solution, Cur-NLC, and different Cur-NAPG-NLC at a dose of 50 mg/kg of Cur, and intravenous injection of Cur solution at a dose of 2 mg/kg of Cur. Data were presented as the mean ± SD (*n* = 6).

The absorption performances of Cur solution, Cur-loaded NLC, and NAPG-NLC in duodenum, jejunum, and ileum of rat were investigated using the in situ single-pass perfusion method. As shown in Figure 4(A,B), the intestinal absorption was enhanced by loading Cur into NLC, since the Ka values were 1.19 and 1.31 fold higher, and P_eff_ values were 1.52- and 1.68-fold higher in duodenum and ileum, respectively, compared with Cur solution (*p* < .05). The Ka and *P*_eff_ values of Cur-NAPG20-NLC showed only 1.20- and 1.62-fold improvement, respectively, in the duodenum, compared with Cur-NLC (*p* < .05). Surprisingly, Cur-NAPG50-NLC and Cur-NAPG100-NLC displayed better absorption from the all intestinal segments compared to Cur-NLC, with the duodenum performing the major absorption. For the absorption of Cur-NAPG50-NLC and Cur-NAPG100-NLC in the duodenum segment, the Ka values were 1.33- and 1.56-fold higher, and the *P*_eff_ values were 1.90 and 2.40 fold higher, respectively, than for Cur-NLC. There was also an obvious increase in permeation of Cur-NAPG20-NLC and Cur-NAPG50-NLC in the jejunum and ileum segments. These results showed that thiolated nanocarriers could improve the intestinal absorption of Cur, and NAPG100-NLC showed better oral absorption potential than NAPG20-NLC and NAPG50-NLC. The increased absorption by intestine has been recognized as a main factor to improve the bioavailability of oral drugs (Tan et al., 2012). The improvement in intestinal absorption of NAPG modified NLC could be explained by following factors. Firstly, Cur was encapsulated in the inner hydrophobic core of NLC, which facilitated the stability and solubility of Cur. Secondly, PEG chains could improve the surface hydrophilicity and impart additional protection to NLC (Tobio et al., 2000). Thirdly, PEG coatings allow NLC to rapidly penetrate into mucus layer by reducing adhesive interactions with mucus (Lai et al., 2011; Yang et al., 2011). Furthermore, the free thiol groups on the surface of Cur-NAPG-NLC can form a disulfide bonds with the cysteine in mucin layer to allow improved interaction of nanoparticles with the mucus layer, thereby increasing the residence time. In addition, thiol groups most likely react with the cell surface by disulfide bonds leading to an absorptive endocytosis, owing to the considerable number of glycoproteins on the surface of epithelial cell (Martien et al., 2007).

## Distribution of NLC and NAPG-NLC in the intestinal tract after oral administration in rats

Intestinal biodistribution of C6-loaded NLC and different NAPG-NLC were investigated by CLSM. As shown in Figure 4(C), the green fluorescence was the strongest in the duodenum after gavage administration in following order: C6-NLC < C6-NAPG20-NLC < C6-NAPG50-NLC < C6-NAPG100-NLC. Obviously, with the increase of the amount of NAPG modification, more green fluorescence could be observed in the duodenum, jejunum, and ileum compared with the plain NLC. These observations indicate that NAPG-modified NLC could rapidly adhere and penetrate through the mucus layer, and then absorbed by enterocytes. The qualitative results by CLSM were in good agreement with the quantitative results obtained by intestinal perfusion.

## Pharmacokinetics

The mean plasma concentration-time curves of Cur after administration of Cur solution, Cur-loaded NLC and different NAPG-NLC at a dose of 50 mg/kg of Cur, and i.v. injection of Cur solution at a dose of 2 mg/kg of Cur, are shown in Figure 4(D), and the pharmacokinetic parameters are listed in Table 2. These results suggest that the oral bioavailability of NLC-based formulation increased 4.27 fold compared with the Cur solution. The reasons for the poor oral bioavailability of Cur are low solubility in the GI fluid and poor drug stability in intestinal or alkaline conditions (Ireson et al., 2002; Anand et al., 2007). Cur exhibits low gut permeability due to rapid metabolism (Berginc et al., 2012), thus, Cur is recognized as a Biopharmaceutics Classification System (BCS) IV class drug (Wahlang et al., 2011). Among the numerous bioavailability enhancement strategies of Cur, nanoparticles were proven effective that do not jeopardize the therapeutic and toxicity profiles of Cur (Anand et al., 2007). The possible reasons for increased oral absorption of Cur-NLC could be the presence of drug in the core thus avoiding the degradation by gastric acid and digestive enzymes and the P-gp drug efflux, and increased drug solubility in nanoparticle form (Müller et al., 1995; Beloqui et al., 2016).

**Table 2.** Pharmacokinetics parameters of Cur after gavage administration of Cur solution, Cur-loaded NLC and NAPG-NLC at a dose of 50 mg/kg of Cur and intravenous injection of Cur solution at a dose of 2 mg/kg of Cur in rats (mean ± SD; *n* = 6).

Pharmacokinetics parameters	Cur solution (p.o.)	Cur solution (i.v.)	Cur-NLC	Cur-NAPG20-NLC	Cur-NAPG50-NLC	Cur-NAPG100-NLC
C_max1_(µg/mL)	0.022 ± 0.006	–	1.05 ± 0.26*	3.76 ± 0.90*^#^	10.45 ± 1.44^△^^□^	20.71 ± 2.40^△^^□^
C_max2_(µg/mL)	–	–	–	3.25 ± 1.34	5.78 ± 1.78	17.36 ± 1.57
AUC_0–10 h_ (µg h /mL)	0.11 ± 0.04	0.17 ± 0.06	0.47 ± 0.21*	8.91 ± 2.83*^#^	16.61 ± 2.48^△^^□^	54.94 ± 3.82^△^^□^
MRT (h)	1.41 ± 0.25	–	1.57 ± 0.08	2.07 ± 0.68*^#^	2.64 ± 0.36^△^^□^	2.90 ± 0.40^△^^□^
CL (L/h)	68.97 ± 3.47	–	20.87 ± 2.75*	1.12 ± 0.11*^#^	0.61 ± 0.24^△^^□^	0.18 ± 0.08^△^^□^
F_Abs _×100	2.60 ± 1.03	100.00	11.01 ± 4.86	209.49 ± 66.48	391.24 ± 58.30	1293.33 ± 89.84

– no data.

* *p* < .05 compared with Cur solution.

△ *p* < .01 compared with Cur solution.

# *p* < .05 compared with Cur-NLC.

□ *p* < .01 compared with Cur-NLC.

As shown in Figure 4(D), the curve of Cur after gavage administration of different Cur-NAPG-NLC and Cur-NLC were much different and NAPG-NLC formulations exhibited superior oral bioavailability profile of Cur. Double absorption peaks were observed in the mean plasma concentration-time profile of Cur-NAPG-NLC as previous reports indicated that Cur might undergo enterohepatic recycling (Ravindranath & Chandrasekhara, 1980; Xiao et al., 2013). NAPG-modified NLC greatly improved the plasma concentration of Cur, with a more prominent bimodal concentration-time curve. NAPG-NLC formulations resulted in the increase of MRT and decrease of clearance when compared with Cur-NLC. It has been reported that the PEG coating improves the surface hydrophilicity and resists plasma protein adsorption, thereby reducing the interaction with phagocytic cells (Shen et al., 2009; Sheng et al., 2009; Jia et al., 2012). Therefore, it is an effective way to increase the circulation time in blood of nanoparticles by PEG modification. The AUC_0–10 h_ of Cur-NAPG20-NLC, Cur-NAPG50-NLC, and Cur-NAPG100-NLC in rats increased 81, 151, and 499.45 folds, respectively, compared to that of Cur solution, and the AUC_0–10 h_ of Cur-NAPG20-NLC, Cur-NAPG50-NLC, and Cur-NAPG100-NLC in rats increased 18.95, 35.34, and 116.89 folds, respectively, compared to that of Cur-NLC. The absolute bioavailability of Cur solution and Cur-NLC was 2.60 and 11.01%, respectively, while the absolute bioavailability of Cur-NAPG-NLC was greater than 100%. This indicates that the modification of NAPG could help to enhance the delivery of NLC into the systemic circulation due to the bioadhesion and permeation-enhancing NAC. In addition, the absorption of Cur increased proportionally with NAPG and is consistent with the results obtained by the intestinal circulation perfusion. Some studies have shown that increasing the thiol content on the surface of nanoparticles can significantly increase the absorption of the drug by improving transmembrane transport of the drug through endocytosis (Bernkop-Schnürch et al., 2004b; Bernkop-Schnürch et al., 2006). Bravo-Osuna et al. (2007) discovered that about 1.5% of mucus cysteine (theoretically about 9.5 mmol SH/g) is present in mucins of the small intestine and are able to form covalent bonds with NAC. Higher density of thiol groups on the surface of NLC could ensure the formation of covalent disulfide bonds with the cysteine groups and might have led to higher bioavailability of NAPG100-NLC than NAPG20-NLC and NAPG50-NLC. NAPG-NLC showed higher bioavailability compared to the plain NLC and we attribute this phenomenon to the multifunctional nanomaterial containing NAC and PEG for enhanced oral absorption of the insoluble drug. The improved absorption of Cur was also facilitated as PEG coatings would have allowed NLC to rapidly penetrate viscoelastic mucus. Subsequently, excellent bioavailability of NAPG-modified NLC was observed *in vivo*. However, mechanism of absorption at the cellular level needs further investigations. In summary, this study has focused on the development of NAPG-NLC to attain suitable bioadhesion and increased permeability for improved oral absorption. The *in vivo* results demonstrate that the intestinal absorption and oral bioavailability of Cur in rats was markedly increased after Cur was encapsulated into NAPG-NLC.

## Conclusions

In this study, a new type of NAPG conjugate was successfully synthesized. This material was used to prepare functional NLC loaded with Cur (NAPG20-NLC, NAPG50-NLC, and NAPG100-NLC) possessing mucoadhesion and mucus penetration properties. Smaller stable nanocarriers possess traits of sustained drug release profile. The intestinal absorption of Cur-NAPG-NLC exhibited notable improvement compared to the simple Cur-NLC further complimented with the CLSM experiments. *In vivo* pharmacokinetic study indicated that AUC of Cur-loaded NAPG-NLC was many folds higher than that of Cur solution and Cur-NLC. In summary, NAPG-modified NLC were superior in performance and own greater potential to improve the oral delivery of BCS IV drugs.

## Supplementary Material

IDRD_Xiao_et_al_Supplemental_Content.doc
