# Supplementary material for: N-acetyl-L-cysteine functionalized nanostructured lipid carrier for improving oral bioavailability of curcumin: preparation, in vitro and in vivo evaluations
Source: Drug Deliv. 2017 Oct 24;24(1):1605–16. doi: 10.1080/10717544.2017.1391890 (PMC8241171; doi:10.1080/10717544.2017.1391890)
Supplement: IDRD_Xiao_et_al_Supplemental_Content.doc [file IDRD_A_1391890_SM5134.doc]

1. **Experimental**

**1.1 Cytotoxicity studies**

Caco-2 cells (human colon cancer cell lines, obtained from the cell bank of Chinese Academy of Sciences) were routinely grown in DMEM medium (KeyGEN BioTECH, Jiangsu, China) with 10% fetal bovine serum, 1% nonessential amino acids and 1% penicillin-streptomycin. Cells were maintained at 37 °C in an incubator (Thermo Electron Corporation) with 5% CO_2_.

The cytotoxicity was evaluated by MTT method. The Caco-2 cells were seeded at a density of 5×10^3^ cells/well in 100 µL DMEM culture media in a 96-well plate at 37 °C and 5% CO_2_. After incubation for 24 h, the DMEM culture media was replaced with media containing different concentrations of blank NLCs (equivalent to 50, 100, 200, 400, 800, and 1600 μg/mL of NLC) and Cur-loaded formulations (equivalent to 0.5, 1, 2, 4, 8, 16 μg/mL of Cur). Meanwhile, serum-free DMEM media incubation without formulations was utilized as a control. After 24 h, 10 µL of 5 mg/mL MTT solution was added into each well for another 4 h, and then the media was exchanged with 150 µL of DMSO. The absorbance was measured at 570 nm with a microplate reader (Thermo Electron Corporation. USA). Cell viability was expressed as a percentage of the absorbance of the study group relative to that of control group.

1. **Results and discussion**

**2.1. Cytotoxicity studies**

The cytotoxicities of blank NLC and different Cur-loaded NAPG-NLCs on Caco-2 cells were investigated using MTT assay. The viability of Caco-2 cells were above 80% for all blank NLCs at the tested concentrations from 50 to 1600 μg/mL of NLC, and no significant difference was found in all formulations, suggesting both blank NLC and different blank NAPG-NLCs might be good biocompatible carriers .

As the concentration of Cur in solution increased from 0.5 to 16 μg/mL, the viability of Caco-2 cells were above 80%, which showed inability of Cur solution to enter the cells and cause significant cell death. However, as the concentration of Cur in the NLC increased from 0.5 to 16 μg/mL, an increase in cellular toxicity was observed in Caco-2 cells. And with the increase of NAPG amount in the NLCs, NAPG-NLC’s toxicity in Caco-2 cells also increased. The IC_50_ of Cur-NAPG20-NLC, Cur-NAPG50-NLC and Cur-NAPG100-NLC were 30.90, 27.11 and 23.94 µg/mL, respectively, and significantly smaller than that of Cur-NLC (47.82 µg/mL) (p<0.05). The increased cytotoxic effect of the Cur-NAPG100-NLC could be attributed to the better penetration of the Cur in Caco-2 cells due to the more NAPG modification.

**2.2. In vitro release study of Cur from different NLC**

Table 1. *In vitro* release kinetics parameters.

| Formulations | Media | Zero-order | | First-order | | Higuchi | | Weibull | | |
| --- | --- | --- | --- | --- | --- | --- | --- | --- | --- | --- |
|  |  | R^2^ | AIC | R^2^ | AIC | R^2^ | AIC | R^2^ | AIC | β ^a^ |
| Cur-NLC | A^b^ | 0.9797 | 22.58 | 0.9823 | 30.23 | 0.9881 | 18.83 | 0.9973 | 9.94 | 1.239 |
|  | B^c^ | 0.9648 | 30.99 | 0.9831 | 33.90 | 0.9831 | 25.14 | 0.9992 | 4.29 | 1.312 |
| Cur-NAPG20-NLC | A^b^ | 0.9972 | 21.93 | 0.9887 | 31.61 | 0.9276 | 49.45 | 0.9991 | 9.15 | 1.110 |
|  | B^c^ | 0.9675 | 24.97 | 0.9929 | 18.99 | 0.9862 | 55.83 | 0.9971 | 9.56 | 0.897 |
| Cur-NAPG50-NLC | A^b^ | 0.9985 | 23.78 | 0.9905 | 34.56 | 0.9404 | 50.87 | 0.9997 | 13.37 | 1.171 |
|  | B^c^ | 0.9842 | 20.63 | 0.9966 | 16.16 | 0.9673 | 52.59 | 0.9996 | 8.43 | 0.983 |
| Cur-NAPG100-NLC | A^b^ | 0.9907 | 20.69 | 0.9762 | 31.72 | 0.8969 | 48.73 | 0.9992 | 7.19 | 1.139 |
|  | B^c^ | 0.9648 | 20.40 | 0.9830 | 17.92 | 0.9831 | 55.13 | 0.9992 | 6.89 | 0.953 |

^a^ β Exponent parameter in Weibull model

^b^ A pH1.2 HCI solution for 2 h and in pH 6.8 PBS for next 22 h

^c^ B physiological saline

**References**

Hu, K., Li, J., Shen, Y., Lu, W., Gao, X., Zhang, Q., et al. (2009). Lactoferrin-conjugated PEG–PLA nanoparticles with improved brain delivery: in vitro and in vivo evaluations*. Journal of Controlled Release, 1*34(1), 55-61.
